# EfficientNetB0 cum FPN Based Semantic Segmentation of Gastrointestinal Tract Organs in MRI Scans

**DOI:** 10.3390/diagnostics13142399

**Published:** 2023-07-18

**Authors:** Neha Sharma, Sheifali Gupta, Mana Saleh Al Reshan, Adel Sulaiman, Hani Alshahrani, Asadullah Shaikh

**Affiliations:** 1 Chitkara University Institute of Engineering and Technology, Chitkara University, Rajpura 140401, Punjab, India; sharma.neha@chitkara.edu.in (N.S.); sheifali.gupta@chitkara.edu.in (S.G.); 2 Department of Information Systems, College of Computer Science and Information Systems, Najran University, Najran 61441, Saudi Arabia; msalreshan@nu.edu.sa (M.S.A.R.); shaikhasad@hotmail.com (A.S.); 3 Department of Computer Science, College of Computer Science and Information Systems, Najran University, Najran 61441, Saudi Arabia; aaalsulaiman@nu.edu.sa; 4 Scientific and Engineering Research Centre, Najran University, Najran 61441, Saudi Arabia

**Keywords:** semantic segmentation, gastrointestinal tract, FPN, PAN, MAnet, Linknet

## Abstract

The segmentation of gastrointestinal (GI) organs is crucial in radiation therapy for treating GI cancer. It allows for developing a targeted radiation therapy plan while minimizing radiation exposure to healthy tissue, improving treatment success, and decreasing side effects. Medical diagnostics in GI tract organ segmentation is essential for accurate disease detection, precise differential diagnosis, optimal treatment planning, and efficient disease monitoring. This research presents a hybrid encoder–decoder-based model for segmenting healthy organs in the GI tract in biomedical images of cancer patients, which might help radiation oncologists treat cancer more quickly. Here, EfficientNet B0 is used as a bottom-up encoder architecture for downsampling to capture contextual information by extracting meaningful and discriminative features from input images. The performance of the EfficientNet B0 encoder is compared with that of three encoders: ResNet 50, MobileNet V2, and Timm Gernet. The Feature Pyramid Network (FPN) is a top-down decoder architecture used for upsampling to recover spatial information. The performance of the FPN decoder was compared with that of three decoders: PAN, Linknet, and MAnet. This paper proposes a segmentation model named as the Feature Pyramid Network (FPN), with EfficientNet B0 as the encoder. Furthermore, the proposed hybrid model is analyzed using Adam, Adadelta, SGD, and RMSprop optimizers. Four performance criteria are used to assess the models: the Jaccard and Dice coefficients, model loss, and processing time. The proposed model can achieve Dice coefficient and Jaccard index values of 0.8975 and 0.8832, respectively. The proposed method can assist radiation oncologists in precisely targeting areas hosting cancer cells in the gastrointestinal tract, allowing for more efficient and timely cancer treatment.

## 1. Introduction

The gastrointestinal (GI) tract aids digestion by breaking down and absorbing food. However, gastrointestinal cancer is a significant public health concern affecting millions globally [1]. Tumors of the esophagus, stomach, large intestine, and small intestine are all examples of GI cancer [2]. The choice of diagnostic method or combination of methods is based on the patient’s symptoms, the suspected condition, and the healthcare provider’s clinical judgment. The accuracy of a diagnosis is essential for the effective treatment and management of diseases. Despite the availability of options such as surgery, chemotherapy, and targeted therapy, radiation therapy has proved to be an effective treatment for GI cancer [3].

Radiation therapy, which employs high-intensity radiation to kill cancer cells, is typically used with other medicines. However, because the GI tract organs are convoluted and irregular in shape, accurate and precise targeting of cancer cells is essential to the success of radiation treatment [4]. Medical diagnostics in GI tract organ segmentation is critical for specific illness detection, multiple diagnosis, appropriate therapy planning, and effective disease monitoring. Diagnostic tests assist in localizing and diagnosing illnesses or anomalies in the GI system by segmenting the organs, allowing for focused treatments and personalized treatment options. Accurate segmentation helps differentiate distinct GI illnesses with similar symptoms, leading to appropriate diagnosis and care. It is critical for detecting the extent and location of conditions, enabling surgical decisions, targeted medicines, and monitoring disease progression or treatment response, all of which contribute to better patient outcomes [5].

Deep learning models have demonstrated significant promise in medical image analysis, notably in organ and structural segmentation [6,7]. This research proposes a hybrid encoder–decoder-based model for semantic segmentation of the GI tract. In the proposed hybrid model, EfficientNet B0 is used as a bottom-up encoder architecture for downsampling to capture contextual information by extracting meaningful and discriminative features from input images. The performance of the EfficientNet B0 encoder is compared with that of three encoders: ResNet 50, MobileNet V2, and Timm Gernet. Here, the Feature Pyramid Network (FPN) is used as a top-down decoder architecture for upsampling to recover spatial information. The performance of the FPN decoder is compared with that of three decoders: PAN, Linknet, and MAnet. Furthermore, the proposed hybrid model is analyzed using Adam, Adadelta, SGD, and RMSprop optimizers. The experiment is carried out utilizing the UW Madison GI tract dataset, which contains 38,496 MRI pictures of cancer patients.

The remaining part of this manuscript is arranged as follows. Section 2 shows the related work for segmenting the GI tract. Section 3 described the input dataset used for the segmentation task. Section 4 shows the proposed methodology for segmenting the small intestine, large intestine, and stomach in MRI images of the UW Madison GI tract dataset. Section 5 depicts the findings of implemented models, and Section 6 concludes the complete manuscript.

## 2. Literature Review

A significant amount of research has been conducted on gastrointestinal tract segmentation and categorization [8,9,10]. Yu et al. developed a unique architecture for polyp identification in the gastrointestinal tract in 2016 [11]. They combine offline and online knowledge to minimize the false acceptance created through offline design and boost recognition results even more. Widespread testing using the polyp segmentation dataset indicated that their solution outperformed others. In 2017, Yuan Y et al. suggested a unique automated computer-aided approach for detecting polyps in colonoscopy footage. They used an unsupervised sparse autoencoder (SAE) to train discriminative features. Then, to identify polyps, a distinctive unified bottom-up and top-down strategy was presented [12]. In 2019, Kang J et al. used the strong object identification architecture “Mask R-CNN” to detect polyps in colonoscopy pictures. They developed a fusion technique to improve results by combining Mask R-CNN designs with differing backbone topologies. They employed three open intestinal polyp datasets to assess the proposed model [13]. In 2019, Cogan T et al. published approaches for enhancing results for a collection of images using full-image pre-processing with a cutting-edge deep learning technique. Three cutting-edge designs based on transfer learning were trained on the Kvasir dataset, and their performance was accessed on the validation dataset. In each example, 80% of the photos from the Kvasir dataset were used to test the model, leaving 20% to validate the model [14]. In 2020, Öztürk et al. developed a successful classification approach for a gastrointestinal tract classification problem. The CNN output is enhanced using a very efficient LSTM structure. To assess the contribution of the proposed technique to the classification performance, experiments were carried out utilizing the GoogLeNet, ResNet, and AlexNet designs. To compare the results of their framework, the same trials were replicated via CNN fusion with ANN and SVM designs [15]. Özturk et al. 2021 presented an artificial intelligence strategy for efficiently classifying GI databases with a limited quantity of labeled images. As a backbone, the proposed AI technique employs the CNN model. Combining LSTM layers yields a categorization. To accurately analyze the suggested residual LSTM architecture, all tests were conducted using AlexNet, GoogLeNet, and ResNet. The proposed technique outperforms previous state-of-the-art techniques [16]. In 2022, Ye R et al. suggested the SIA-Unet, an upgraded Unet design that utilizes MRI data. It additionally contains an attention module that filters the spatial information of the feature map to fetch relevant data. Many trials on the dataset were carried out to assess SIA-Unet’s performance [17]. In 2022, Nemani P et al. suggested a hybrid CNN–transformer architecture for segmenting distinct organs from images. With Dice and Jaccard coefficients of 0.79 and 0.72, the proposed approach is resilient, scalable, and computationally economical. The suggested approach illustrates the principle of deep learning to increase treatment efficacy [18]. Chou, A. et al. used U-Net and Mask R-CNN approaches to separate organ sections in 2022. Their best U-Net model had a Dice score of 0.51 on the validation set, and the Mask R-CNN design received a Dice value of 0.73 [19]. In 2022, Niu H et al. introduced a technique for GI tract segmentation. Their trials used the Jaccard index as the network assessment parameter. The greater the Jaccard index, the better the model. The results demonstrate that their model improves the Jaccard index compared to other methods [20]. In 2022, Li, H, and colleagues developed an improved 2.5D approach for GI tract image segmentation. They investigated and fused multiple 2.5D data production methodologies to efficiently utilize the association of nearby pictures. They suggested a technique for combining 2.5D and 3D findings [21]. In 2022, Chia B et al. introduced two baseline methods: a UNet trained on a ResNet50 backbone and a more economical and streamlined UNet. They examined multi-task learning using supervised (regression) and self-supervised (contrastive learning) approaches, building on the better-performing streamlined UNet. They discovered that the contrastive learning approach has certain advantages when the test distribution differs significantly from the training distribution. Finally, they studied Featurewise Linear Modulation (FiLM), a way of improving the UNet model by adding picture metadata such as the position of the MRI scan cross-section and the pixel height and breadth [22]. Georgescu M. et al. suggested a unique technique for generating ensembles of diverse architectures for medical picture segmentation in 2022 based on the variety (decorrelation) of the models constituting the ensemble. They used the Dice score among model pairs to measure the correlation between the outputs of the two models that comprise each pair. They chose models with low Dice scores to foster variety. They conducted gastrointestinal tract image segmentation studies to compare their diversity-promoting ensemble (DiPE) with another technique for creating ensembles that relies on picking the highest-scoring U-Net models [23].

## 3. Input Dataset

**Figure 1:**
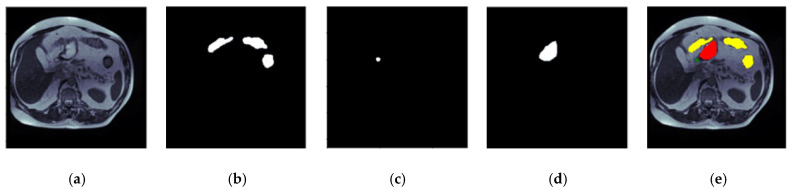
UW Madison GI tract dataset, (**a**) input image mask, (**b**) large bowel mask, (**c**) small bowel mask, (**d**) stomach mask, and (**e**) concatenated mask with large bowel in yellow color, small bowel in green color and stomach in red color [24].

This research employs magnetic resonance imaging (MRI) data collected from patients who underwent MRI-guided radiotherapy at the University of Wisconsin-Madison Carbone Cancer Center. This research uses a dataset comprising 85 patients, encompassing 38,496 scans of various GI parts. The 16-bit grayscale Portable Network Graphics (PNG) layout represents the scans, while the annotations are given in comma-separated values (CSV) representations. The ground truth mask is generated from these annotations using an RLE encoder. Hence, there are 14,085 masks for the large bowel, 11,201 masks for the small bowel, and 8627 masks for the stomach. A number of 33,913 masks do not include any organs from the GI tract, so these are blank masks. The RLE-encoded masks are used to describe the segmented areas. The dataset is available on the Kaggle website [24]. The dimensions of each slice exhibit variability, ranging from 234 × 234 to 384 × 384 pixels. Figure 1 shows an image of the dataset with its ground truth masks. Figure 1a shows the input image of case32_day19_slice_0089. Figure 1b shows the mask for the large bowel, Figure 1c shows the small bowel, Figure 1d shows the mask for the stomach, and Figure 1e shows an image with three concatenated masks.

## 4. Proposed Methodology

**Figure 2:**
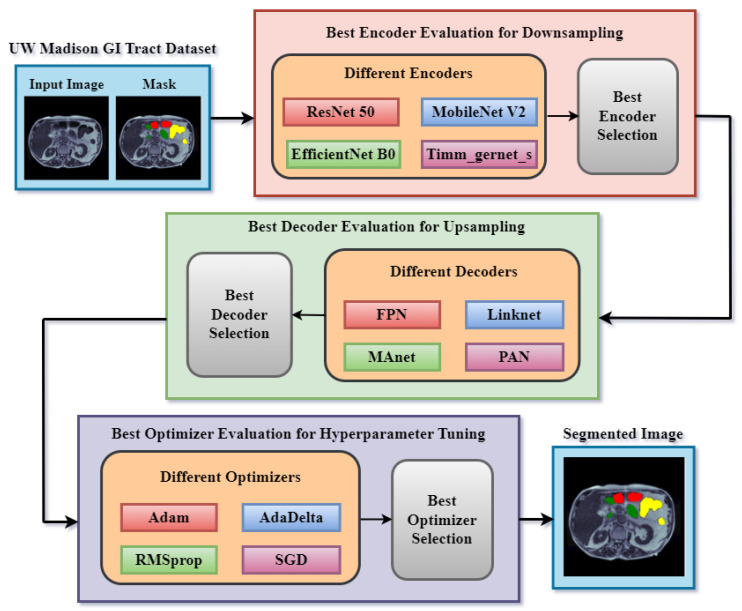
Proposed methodology.

This research presents a segmentation model for segmenting GI tract parts such as the stomach and small and large bowel. Figure 2 depicts the proposed technique, which includes the input dataset, which is the UW Madison GI tract dataset. The second block is a downsampling encoder. Several encoders are used for downsampling in semantic segmentation to derive meaningful and hierarchical representations from the input data. To discover the optimum encoder for the segmentation job, four different encoders are implemented: ResNet 50 [25], EfficientNet B0 [26], MobileNet V2 [27], and Timm Gernet [28]. These encoders are pre-trained transfer learning models that did well on the ImageNet dataset. These encoders play a vital role in downsampling the input data, allowing the decoder network to construct accurate and complete semantic segmentation maps of the gastrointestinal system. Different performance measures are used to assess these encoders. The best encoder will then be finalized based on the results and utilized as the encoder component of the final optimized model.

Several decoders are used for upsampling in semantic segmentation to regain spatial resolution and construct high-resolution segmentation maps. Upsampling is required because it restores the fine-grained details lost during downsampling. Dilated convolution-based decoders maintain spatial resolution while increasing the receptive field. By varying the dilation rates in the decoder, these devices successfully capture fine features and contextual information at several scales. The sort of decoder employed is decided by the application’s specific requirements and the nature of the target objects. Some decoders are better at capturing little details, while others may be better at maintaining spatial context. Four alternative decoders are used to determine the optimum decoder for GI tract segmentation. The Feature Pyramid Network (FPN) [29], Pyramid Attention Network (PAN) [30], Linknet [31], and MAnet [32] are the names of the four decoders. These segmentation models were chosen for their excellent performance in earlier medical imaging research and their versatility in dealing with characteristics of various sizes. The best decoder is selected based on the findings of these four models.

Optimizers for hyperparameter tuning are additional components of the proposed technique. Semantic segmentation employs several optimizers to improve training efficacy and subsequent model performance. Several variables impact the selection of which optimizer to utilize, including the dataset, model design, available computational resources, and the demands of the segmentation task. In this case, four different optimizers are evaluated: Adam [33], Adadelta [34], RMSprop [35], and SGD [36]. The best optimizer is chosen based on the results obtained by several optimizers. After the encoder, decoder, and optimizer selection experiments, the most optimized model will be finalized. The final model will partition the input picture into three classes: small bowel, big colon, and stomach. In both the mask and the segmented image, yellow represents the big intestine, green represents the small colon, and red represents the stomach.

## 5. Results and Discussions

This section displays the results of the different encoder, decoder, and optimizer evaluations. We used the Google Colab platform, Keras and TensorFlow environments, and the Python programming language for the experiments.

### 5.1. Encoder Evaluation for Downsampling

**Figure 3:**
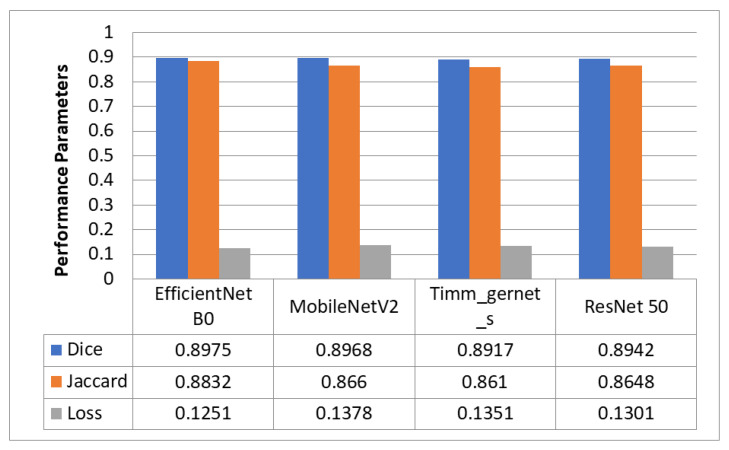
Comparison of Dice and Jaccard coefficients and loss of different encoders.

**Figure 4:**
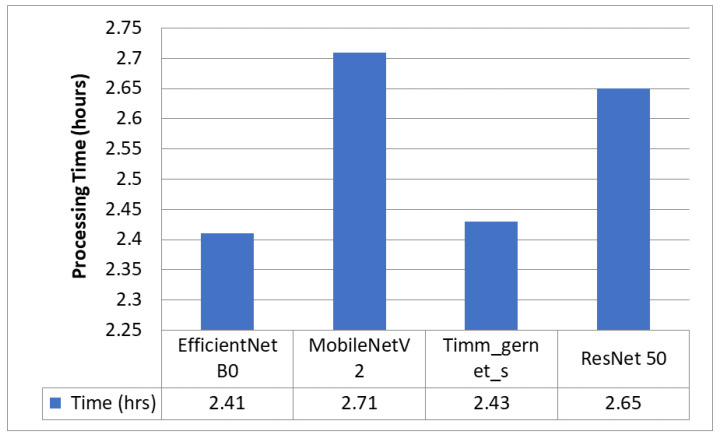
Processing time comparison for different encoders.

Figure 3 compares four encoders that segment GI organs in the GI tract using the Dice coefficient, Jaccard coefficient, and loss. The four encoders are EfficientNet B0, MobileNet V2, Timm_Gernet_S, and ResNet 50. Figure 4 compares different encoders in terms of the processing time required by each encoder model. The findings reveal that EfficientNet B0 had the most significant Dice coefficient of 0.8975 and Jaccard coefficient of 0.8832, with a loss of 0.1251 and the shortest processing time of 2 h and 25 min. MobileNetV2 likewise performed well, with a Dice coefficient of 0.8968, a Jaccard coefficient of 0.866, and a loss of 0.1378, but needed slightly more processing time than EfficientNet B0. Timm_gernet_s obtained a Dice coefficient of 0.8917, a Jaccard coefficient of 0.8610, and a loss of 0.1351 in 2 h and 26 min. ResNet 50 had the same Dice and Jaccard coefficients as Adam, with a loss of 0.1301 and a processing time of 2 h and 39 min. In conclusion, the results indicate that EfficientNet B0 is the most effective encoder model for segmenting GI organs in the GI tract.

### 5.2. Best Encoder—EfficientNet B0

The EfficientNet-B0 architecture has become a well-known convolutional neural network (CNN) architecture suitable for use as an encoder in semantic segmentation tasks. EfficientNet-B0 was used in the proposed research design as a backbone network to extract features from the input image using downsampling. The current study proposes a unique network design using a compound scaling strategy. A very accurate and efficient model is produced by this approach, which balances the network’s depth, breadth, and resolution.

**Figure 5:**
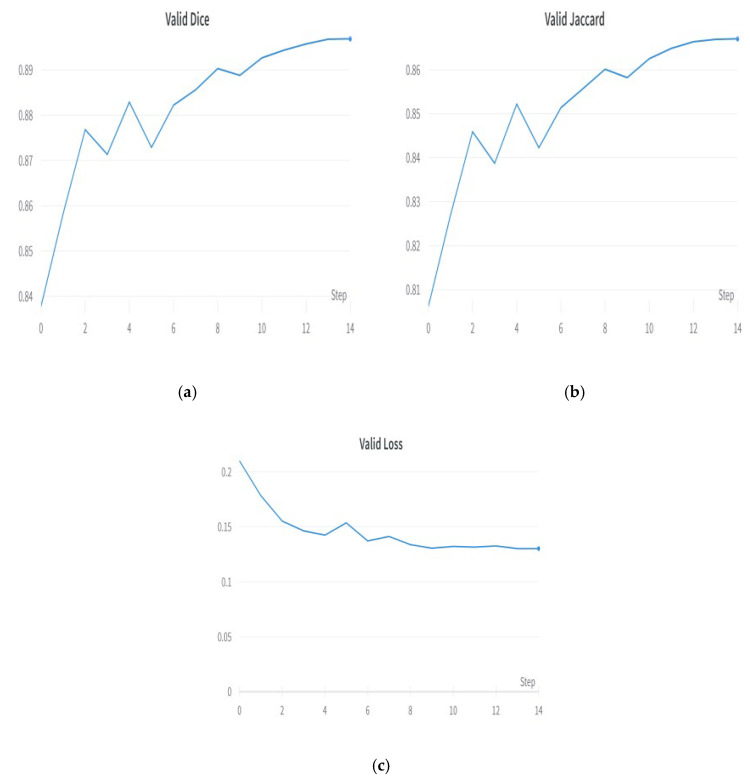
Results with best encoder—EfficientNet B0: (**a**) validation Dice coefficient, (**b**) validation Jaccard coefficient, and (**c**) validation loss.

EfficientNet-B0 is a convolutional neural network architecture composed of multiple blocks, each incorporating a blend of convolutional layers, activation functions, and pooling operations. It is a convolutional neural network architecture widely used for image classification tasks. In the context of semantic segmentation, the output of EfficientNet-B0 is commonly utilized as input to a decoder network. Using EfficientNet-B0 as an encoder for semantic segmentation has resulted in exceptional levels of accuracy and efficiency across a range of applications, including medical image segmentation [26]. Figure 5 shows the plots of the encoder model. Figure 5a shows the validation Dice coefficient, Figure 5b shows the validation Jaccard coefficient, and Figure 5c shows the model loss plot. EfficientNet B0 outperformed other encoders, such as ResNet 50, MobileNet V2, and Timm Gernet.

### 5.3. Decoder Evaluation for Upsampling

**Figure 6:**
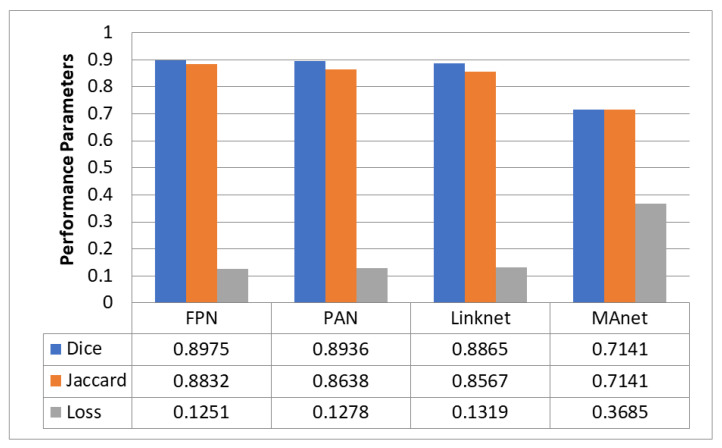
Comparison of Dice and Jaccard coefficient and loss of different decoders.

**Figure 7:**
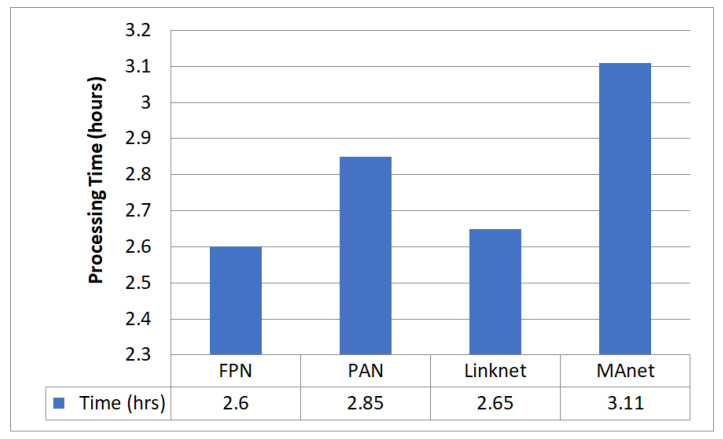
Comparison of processing time required for different decoders.

Figure 6 compares the four decoders used to segment GI organs in the GI tract using the Dice coefficient, Jaccard coefficient, and loss. The names of the four decoders used are FPN, PAN, LinkNet, and MAnet. Figure 7 compares the different decoders in terms of the processing time required by each decoder model. FPN had the most significant Dice coefficient of 0.8975 and Jaccard coefficient of 0.8832, with a loss of 0.1251 and a processing time of 2 h and 39 min. PAN fared similarly to FPN, with a Dice coefficient of 0.8936, a Jaccard coefficient of 0.8638, and a loss of 0.1278. It took significantly longer to process. Linknet produced a Dice coefficient of 0.8865, a Jaccard coefficient of 0.8567, and a loss of 0.1319 in 2 h and 36 min. MAnet, on the other hand, had the lowest Dice and Jaccard coefficients and the most significant loss, with a Dice and Jaccard coefficient of 0.7141 and a loss of 0.3685. MAnet also needed the most processing time (3 h and 7 min). Finally, the results indicate that FPN is the most successful segmentation model for segmenting GI organs in the GI tract.

### 5.4. Best Decoder—FPN

**Figure 8:**
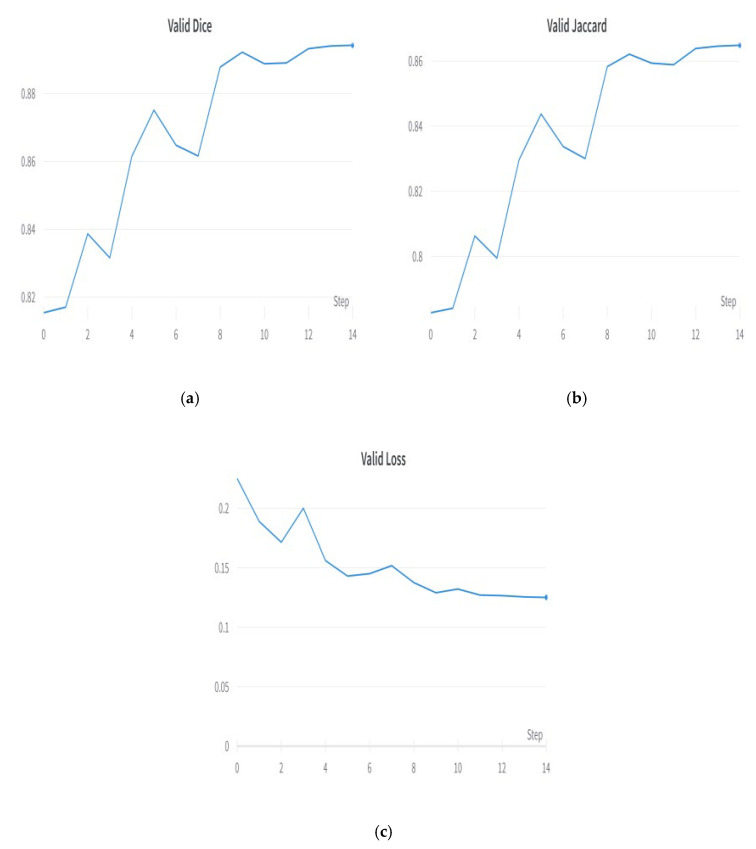
Result with best decoder—FPN: (**a**) validation Dice coefficient, (**b**) validation Jaccard coefficient, and (**c**) validation loss.

The FPN segmentation model is a famous deep learning architecture used for medical picture segmentation and other semantic segmentation problems. The FPN segmentation model’s structure entails a segmentation head, a top-down pathway, lateral connections, and a backbone network. After several upsampling and convolutional layers, the top-down route produces feature maps with varying spatial resolutions. The feature maps from the top-down pathway are linked to the feature maps from the backbone network through lateral connections. Because of this, the model can accurately represent details across several scales. The segmentation head then uses the fused feature maps to predict the segmentation masks for the various item classes in the input picture. As a result of its well-designed architecture, the FPN segmentation model is widely used in a wide variety of picture segmentation tasks [29]. Figure 8 shows the plots of the FPN segmentation model. Figure 8a shows the validation Dice coefficient, Figure 8b shows the validation Jaccard coefficient, and Figure 8c shows the model loss plot. The FPN outperformed decoders such as PAN, Linknet, and MAnet.

### 5.5. Optimizer Evaluation for Hyperparameter Tuning

**Figure 9:**
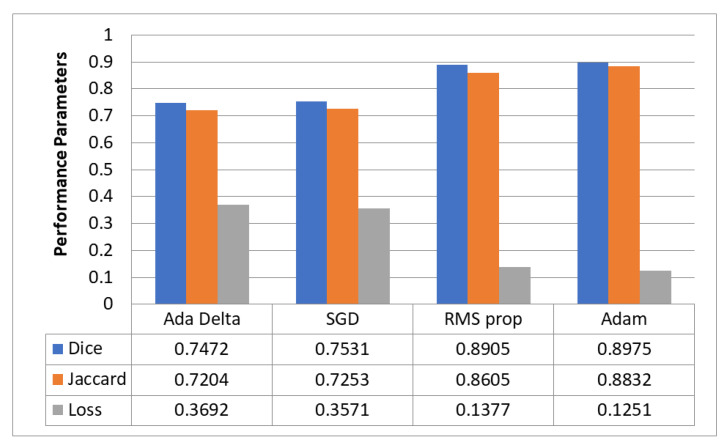
Comparison of Dice and Jaccard coefficients and loss of different optimizers.

**Figure 10:**
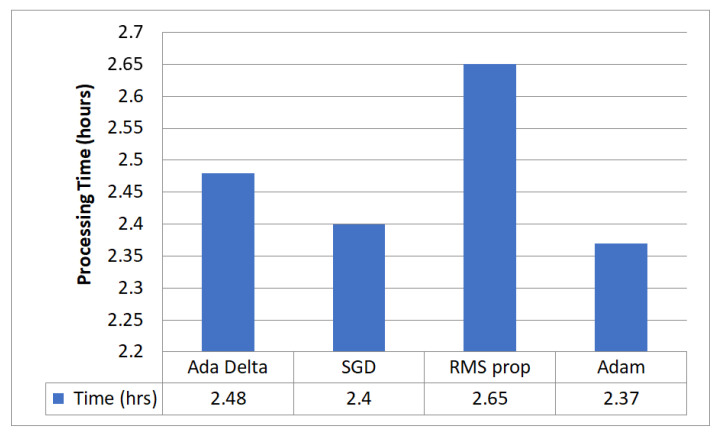
Comparison of processing time required for different optimizers.

Figure 9 evaluates the performance of the proposed model with four optimizers that segment GI organs in the GI tract using the Dice coefficient, Jaccard coefficient, and loss. Figure 10 compares different optimizers regarding the processing time required by the proposed model. The findings reveal that the Adam optimizer obtained the most significant Dice coefficient of 0.8975 and Jaccard coefficient of 0.8832, with the lowest loss of 0.1251. Adam needed 2 h and 28 min to complete the processing. The RMS prop also performed well, with a Dice coefficient of 0.8905, a Jaccard coefficient of 0.8605, and a loss of 0.1377. However, it took a little longer to digest than Adam. SGD and Ada Delta, on the other hand, achieved a worse Dice and Jaccard coefficient performance and more significant loss than the other optimizers. SGD had a Dice coefficient of 0.7531 and a Jaccard value of 0.7253, with a loss of 0.3571, whereas Ada Delta had a Dice coefficient of 0.7472, a Jaccard coefficient of 0.7204, and a loss of 0.3692. In conclusion, the results indicate that Adam is the most effective optimizer for segmenting GI organs in the GI tract.

### 5.6. Best Optimizer—Adam

**Figure 11:**
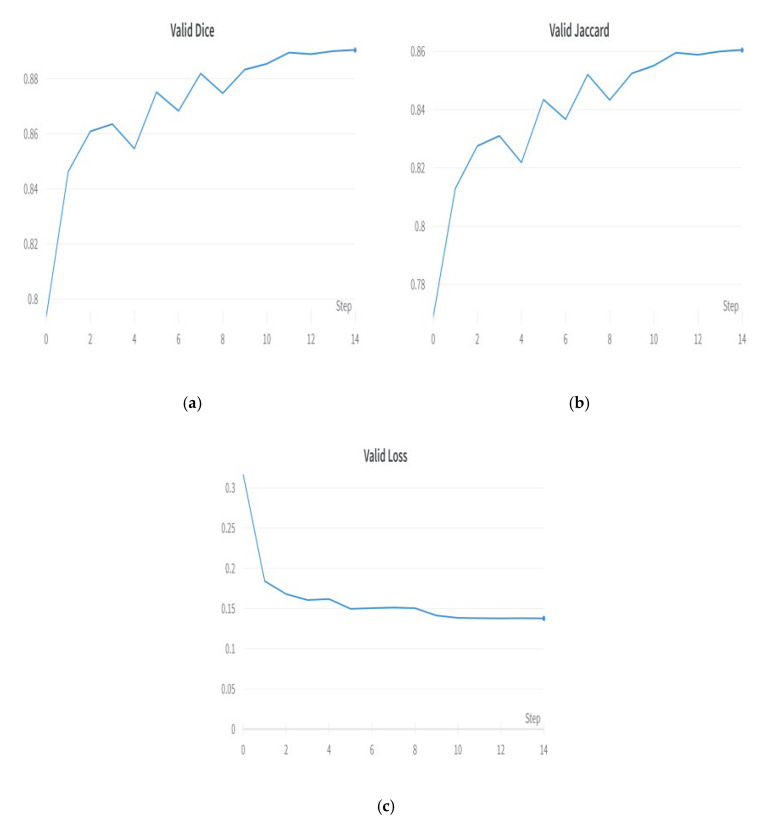
Results with best optimizer—Adam: (**a**) validation Dice coefficient, (**b**) validation Jaccard coefficient, and (**c**) validation loss.

The Adam optimizer is a common choice for training deep neural networks for semantic segmentation problems. Adam stands for “Adaptive Moment Estimation”, being an adaptation of the stochastic gradient descent (SGD) optimizer that employs adaptive learning rates for each weight parameter in the network [33]. Adam operates in semantic segmentation by modifying the learning rate for each weight parameter based on its first and second moments. This adaptive learning rate modification leads to faster convergence and better optimization performance than classic gradient-descent-based optimizers. Adam can also handle sparse gradients, which is helpful for segmentation jobs in which many pixels have no labels. The optimizer’s hyperparameters, such as learning rate and momentum, may be modified to optimize segmentation performance on a given dataset. Adam is a popular choice for semantic segmentation problems because of its quick convergence, variable learning rate modification, and capacity to handle sparse gradients. Figure 11 shows the plots of the Adam optimizer. Figure 11a shows the validation Dice coefficient, Figure 11b shows the validation Jaccard coefficient, and Figure 11c shows the model loss plot. The Adam optimizer outperformed other optimizers, such as AdaDelta, RMSprop, and SGD.

### 5.7. Visualization of Results for the Best Optimized Model

**Figure 12:**
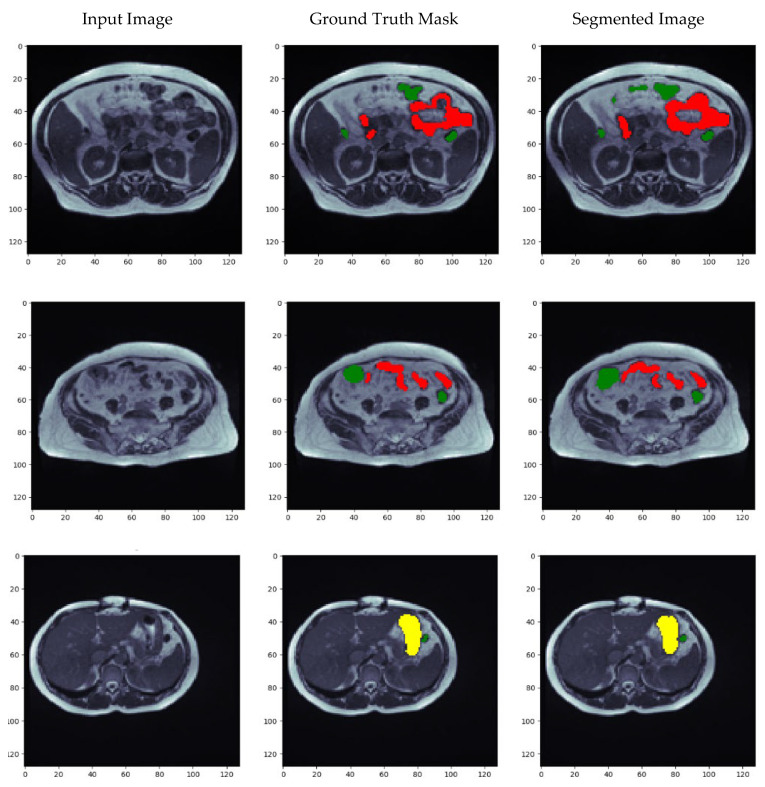

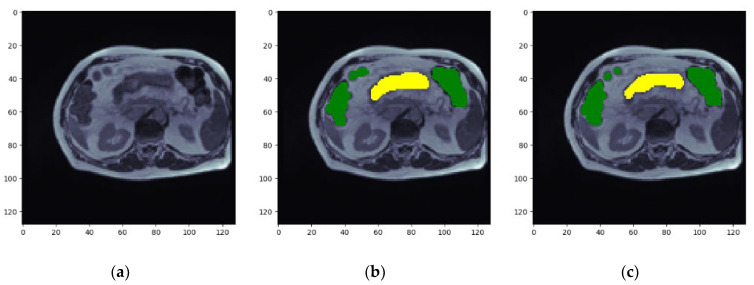
Visualization of results (**a**) Input images, (**b**) Ground truth masks and (**c**) Segmented Image with large bowel in yellow color, small bowel in green color and stomach in red color.

Figure 12 depicts the results of the model in the form of images. Figure 12 includes the input image, ground truth mask, and the predicted segmented image. Here, yellow represents the large bowel, green is for the small bowel, and red is for the stomach. The similarity between the ground truth mask and the segmented image shows how much the proposed method can accurately segment the input image. It can be seen in the images that the segmented images are very similar to the ground truth mask of the input image. Thus, the proposed model can segment MRI scans of the gastrointestinal tract to assist radiation therapy to speed up the treatment.

## 6. State-of-the-Art Comparison of UW Madison GI Tract Dataset

Table 1 summarizes several approaches and their associated outcomes for the segmentation of GI tract organs using the UW Madison GI tract dataset. The references and years of publication are provided, and the procedures utilized and the findings obtained are mentioned in Table 1. In 2022, the SIA UNet method received a Dice score of 0.78. The CNN Transformer obtained a somewhat higher Dice score of 0.79 and an IoU score of 0.72. The combination of UNet and Mask RCNN yielded a Dice score of 0.51. Furthermore, Unet, when used on 2.5D data, produced a Dice score of 0.36% and an IoU score of 0.12%. An ensemble of multiple architectures performed well, with a Dice score of 0.88. Finally, the proposed model, a hybrid EfficientNet B0 combined with an FPN, received the highest Dice score of 0.8975 and an IoU score of 0.8832. Table 1 reveals that the proposed model outperformed the state-of-the-art techniques for the UW Madison GI tract dataset in segmenting GI tract organs.

## 7. Conclusions

The gastrointestinal tract (GI) is a critical mechanism in the human body that aids nutrition, digestion, and absorption. It breaks down food into smaller molecules that the body can absorb and utilize. There has been a significant increase in GI malignancies among men and women in recent years. Radiation therapy is usually considered the most common treatment for GI cancer. The therapy includes applying high-energy X-rays to target malignant cells while avoiding healthy organs in the GI system. Therefore, it is essential to develop an automated method for accurately segmenting GI tract organs to speed up medical therapy. Medical diagnosis in GI tract organ segmentation has various advantages. Accurate segmentation of GI organs enables accurate illness detection and localization, assisting in early diagnosis and tailored therapy planning. This research proposes a hybrid encoder–decoder-based model for semantic segmentation of the GI tract. In the proposed hybrid model, EfficientNet B0 is used as a bottom-up encoder architecture for downsampling to capture contextual information by extracting meaningful and discriminative features from input images.

In contrast, the Feature Pyramid Network (FPN) is a top-down decoder architecture used for upsampling to recover spatial information. The proposed model achieved Dice coefficient and Jaccard index values of 0.8975 and 0.8832, respectively. This research aimed to find the most feasible combination of these components for segmentation optimization. In this study, the best-performing model used EfficientNet B0 as the encoder, FPN as the decoder, and Adam as the optimizer. This strategy is likely to improve cancer therapy efficacy and timeliness.

## Figures and Tables

**Table 1:** State-of-the-art comparison.

Ref/Year	Techniques	Dice	IoU/Jaccard
[17]/2022	SIA UNet	0.78	-
[18]/2022	CNN Transformer	0.79	0.72
[19]/2022	UNet and Mask RCNN	0.51	-
[20]/2022	UNet on 2.5D	0.36	0.12
[21]/2022	Ensemble of Different Architectures	0.88	-
[37]/2022	UNet	0.8854	0.8819
Proposed Model	EfficientNetB0 and FPN	0.8975	0.8832

## Data Availability

The dataset is available on the Kaggle website https://www.kaggle.com/competitions/uw-madison-gi-tract-image-segmentation/data accessed on 8 February 2023.
